# Membrane Binding of Plasmid DNA and Endocytic Pathways Are Involved in Electrotransfection of Mammalian Cells

**DOI:** 10.1371/journal.pone.0020923

**Published:** 2011-06-13

**Authors:** Mina Wu, Fan Yuan

**Affiliations:** Department of Biomedical Engineering, Duke University, Durham, North Carolina, United States of America; Stanford, United States of America

## Abstract

Electric field mediated gene delivery or electrotransfection is a widely used method in various studies ranging from basic cell biology research to clinical gene therapy. Yet, mechanisms of electrotransfection are still controversial. To this end, we investigated the dependence of electrotransfection efficiency (eTE) on binding of plasmid DNA (pDNA) to plasma membrane and how treatment of cells with three endocytic inhibitors (chlorpromazine, genistein, dynasore) or silencing of dynamin expression with specific, small interfering RNA (siRNA) would affect the eTE. Our data demonstrated that the presence of divalent cations (Ca^2+^ and Mg^2+^) in electrotransfection buffer enhanced pDNA adsorption to cell membrane and consequently, this enhanced adsorption led to an increase in eTE, up to a certain threshold concentration for each cation. Trypsin treatment of cells at 10 min post electrotransfection stripped off membrane-bound pDNA and resulted in a significant reduction in eTE, indicating that the time period for complete cellular uptake of pDNA (between 10 and 40 min) far exceeded the lifetime of electric field-induced transient pores (∼10 msec) in the cell membrane. Furthermore, treatment of cells with the siRNA and all three pharmacological inhibitors yielded substantial and statistically significant reductions in the eTE. These findings suggest that electrotransfection depends on two mechanisms: (i) binding of pDNA to cell membrane and (ii) endocytosis of membrane-bound pDNA.

## Introduction

Pulsed electric field has been widely used for many years for improving gene delivery into cells both *in vitro* [1], [2] and *in vivo* [3], [4], [5], [6], [7], [8], [9], [10]. The technique is considered to rely on transient permeabilization of the plasma membrane of cells at hyperpolarized and depolarized poles facing the anode and cathode [11], [12], respectively, to allow polyanionic plasmid DNA (pDNA) to enter cells through permeabilized membrane facing the cathode. Several different terms have been used to describe the technique, including electroporation, electropermeabilization, electrogene transfer, gene electroinjection, and electrotransfection [13]. These alternative terms are referred to as electrotransfection in this paper. Despite its numerous applications in biology, the main disadvantage of this technique, compared to other gene delivery methods, is the difficulty in controlling its efficiency, which can vary by several orders of magnitude under different experimental conditions and electric field parameters. The optimization of cell transfection proceeds primarily by trial and error because of the poor understanding of the mechanisms governing electrotransfection.

It has been widely accepted that electrotransfection is dependent upon the phenomenon known as electroporation, whereby transient, hydrophilic pores are generated in the plasma membrane when the electric field-induced transmembrane potential difference exceeds a certain threshold level (200–1000 mV) [14]. Cell-impermeant molecules are then transported through these pores via mechanisms that may include diffusion [15], electrophoresis [3], and electroosmosis [16]. These mechanisms are likely to apply for delivery of small molecules but have yet to be shown to facilitate DNA transport across the membrane [12], [13], [16], [17], [18], [19], [20], [21]. More recently, emerging evidence from various studies is challenging the “electroporation” mechanism for gene delivery [22], [23], [24]. Golzio *et al.* directly visualized electric field-mediated cell entry of pDNA in an *in vitro* study [23]. Their observations in this and follow up studies demonstrate that applied electric field induces complex formation between pDNA and plasma membrane and that translocation of these complexes through the membrane occurs after, rather than during, electric pulse application [22], [23], [24]. The implication of these studies is that the applied electric field is necessary for electrophoretically pushing pDNA toward the cell membrane and for initiating complex formation between pDNA and the cell membrane, but that it may not be a driving force for pDNA entry into the cytosol. Therefore, the questions remain as to what are mechanisms of pDNA internalization and how is it regulated by cells?

Another important observation in the literature is that DNA fragments of sizes comparable to pDNA are largely immobilized after direct injection into the cytosol [25], [26], indicating that diffusion is highly improbable as a dominant mode of pDNA transport in the cytosol. The hindered diffusion has been attributed to cytoplasmic crowding posed by the presence of various organelles, high protein concentrations, and highly cross-linked network of actin filaments [27]. The cytosolic diffusional barrier is further exacerbated by the short half-life of naked pDNA, due to degradation by intracellular nucleases. The half-life of DNA in the cytosol is 1–2 hr in HeLa and COS-1 cells [28] and only 5 min in muscle cells [29], suggesting that the time window for intracellular diffusion of intact pDNA is short. The short time window and diffusional barriers imply that most internalized pDNA molecules cannot reach the nuclear envelope via diffusion [30], [31]. How, then, can electrotransfection achieve the high efficiencies observed in some studies? What are the mechanisms of intracellular transport?

To answer the questions raised above, we investigated dynamics of electric field-induced pDNA interactions with the cell membrane and subsequent pDNA internalization and intracellular transport. Data from the study revealed that electrotransfection strongly relies upon (i) binding of pDNA to plasma membrane during electric field exposure and (ii) internalization of the membrane bound pDNA via endocytic-like processes.

## Results

### Effects of divalent cations on pDNA adsorption to cell membrane and electrotransfection

**Figure 1:**
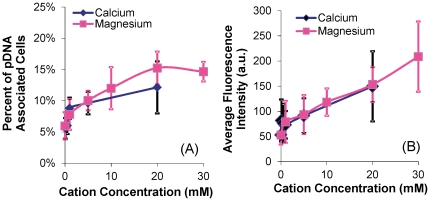
Dependence of membrane-bound pDNA on cation concentrations. pDNA was labeled with YOYO-1 dye with basepair-to-dye ratio of 5∶1. The binding was characterized in terms of (A) percent of pDNA-associated cells and (B) average fluorescence intensity with arbitrary unit (a.u.) per pDNA-associated cell. The number of independent trials (n) was 5–6. The symbols and error bars denote means and standard deviations, respectively.

**Figure 2:**
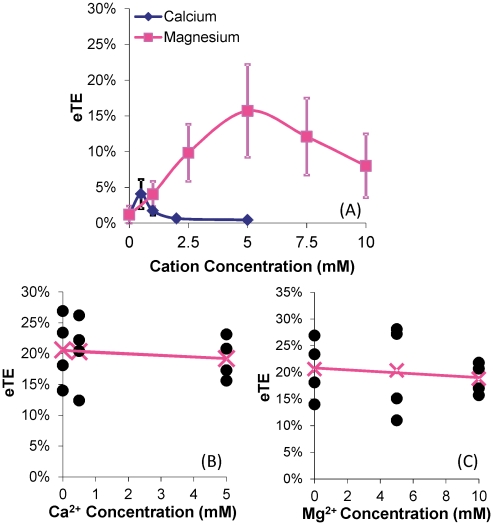
Dependence of electrotransfection efficiency on cation concentrations. eTE is defined as the percent of live cells expressing GFP. B16-F10 cells were electrotransfected (400 V/cm, 5 msec, 8 pulses, 1 Hz) with unlabeled GFP-encoding pDNA in a transfection buffer. GFP expression was measured using flow cytometry after 24 hr incubation. (A) The low ionic strength medium supplemented with Ca^2+^ or Mg^2+^ at varying concentrations was used as the electrotransfection buffer. n = 7–8. The symbols and error bars denote means and standard deviations, respectively. The peak eTE value in each curve was significantly higher than those at both ends of the same curve (P<0.05). In Panels (B) and (C), OptiMEM was used as the electrotransfection buffer. After 20 min incubation post electrotransfection, the cells were re-suspended in the low ionic strength medium supplemented with either Ca^2+^ or Mg^2+^ at varying concentrations and treated again with the same electric field. The GFP expression was quantified at 24 hr. n = 4. The filled circles denote data from individual samples, the “x” symbol represents the mean of the samples at a given cation concentration, and the line represents the linear regression of the mean data. The mean value was statistically independent of the variation in Ca^2+^ and Mg^2+^ concentrations (P>0.05, Mann Whitney U test).

It has been reported that pulsed electric field induces complex formation between pDNA and cell membrane [22], [23], [24]. Although mechanisms of this complex formation are still unknown, we hypothesized that the process could be facilitated by divalent or multi-valent cations that cross-link and, hence, anchor pDNA to negatively charged carbohydrates and proteins on the plasma membrane. To test the hypothesis, we investigated the effects of divalent cations on pDNA binding to B16.F10 cell membrane, and quantified the dependence of pDNA adsorption on concentrations of Ca^2+^ and Mg^2+^. In the study, fluorescently labeled pDNA was mixed with cells suspended in various cation-supplemented buffers and left to incubate for 10 min on ice and 10 min at the room temperature to promote adsorption, before flow cytometric analysis (for details see the Materials and Methods section). It was observed that the percentage of pDNA-associated cells was a nonlinear function of the concentration for each cation (see **Figure 1A**), reaching a maximum value of ∼16%. The average intensity of the pDNA-associated cells, on the other hand, increased approximately linearly with no sign of saturation when the concentration of each cation was increased (see **Figure 1B**). To investigate the subsequent effects of this divalent cation-mediated pDNA adsorption on the electrotransfection of a reporter gene encoding green fluorescence protein (GFP), the cells were incubated with pDNA in the various cation-supplemented buffers for 10 min on ice to promote adsorption, followed by exposure to an electric field (8 pulses at 400 V/cm, 5 msec duration, and 1 Hz frequency) at the room temperature. After 24 hr of incubation, electrotransfection efficiency (eTE) was quantified using flow cytometry. The data shown in **Figure 2A** demonstrated that the eTE was enhanced by Ca^2+^ and Mg^2+^ at low concentrations but the enhancement was reduced when the concentrations were further increased. When no divalent cations were present in the low ionic strength buffer, the eTE was close to zero, suggesting that (a) cation-mediated pDNA adsorption was a necessary step for cell transfection and (b) electrophoresis of the polyanionic pDNA, which is inversely proportional to the ionic strength of a solution, did not play a critical role in electrotransfection, as also suggested by some previous studies [32]. To eliminate the possibility that the cation-dependent nature of eTE might be attributed to other cation-induced cellular effects aside from mediating pDNA-membrane interactions (i.e. changes in transcriptional and translational activities), a control experiment was performed in which cells were first electrotransfected with pDNA in OptiMEM. After a 20 min incubation period to permit sufficient membrane recovery, the cells were re-suspended in the low ionic strength buffer supplemented with cations at varying concentrations. The suspension was exposed again to the same electric field as before. The results shown in **Figures 2B and 2C** demonstrated that when cations were not present during the actual pDNA electrotransfection but rather, introduced into the cells in the presence of electric field 20 min later, the varying cation concentrations did not produce statistically significant changes in eTE, suggesting that Ca^2+^ and Mg^2+^ had minimal effects on normal transcriptional and translational activities in treated cells.

### Slow internalization of pDNA after exposure to electric field

**Figure 3:**
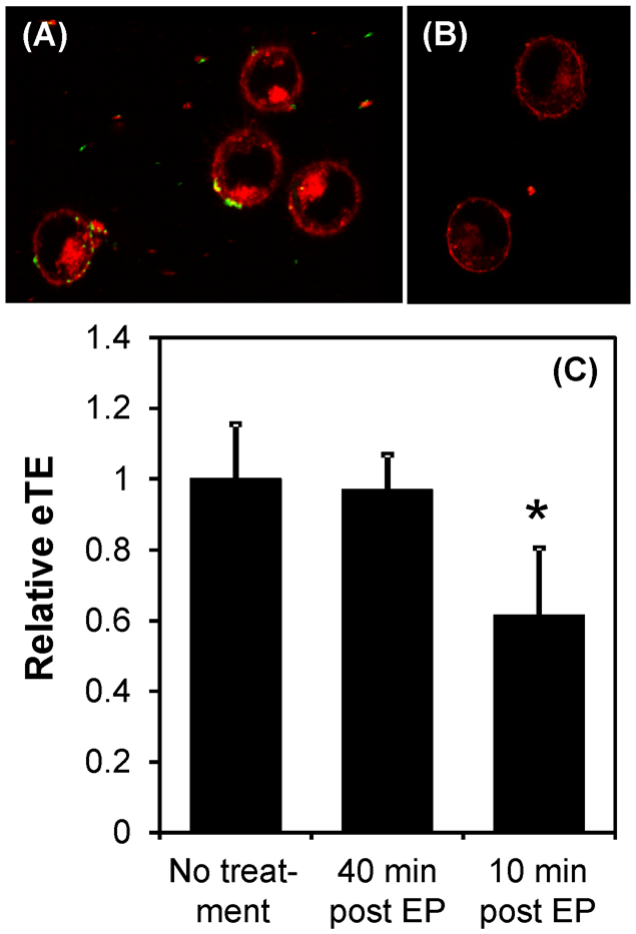
Effects of trypsin treatment on pDNA adsorption to cell membrane and eTE. (A) YOYO 1-labeled pDNA (green) formed complexes with FM4-64FX labeled plasma membrane (red) following exposure of cells to pulsed electric field (400 V/cm, 5 msec, 8 pulses, 1 Hz). The image was taken shortly after the application of electric field. (B) The experimental protocol was the same as that in the Panel (A), except that at 10 min post electric field exposure, the cells were treated with 0.25% trypsin-EDTA solution for 30 min at 37°C. The image was taken after the trypsin treatment. (C) B16-F10 cells in pDNA solution were exposed to the same electric pulses (EP) as above. At 10 or 40 min post EP exposure, the cells were treated with 0.25% trypsin-EDTA for 30 min at 37°C. Then, the cells were cultured for 24 hr at 37°C. The eTE was measured as the percent of live cells expressing GFP and normalized by the data from the untreated group. The solid column and error bar represent mean and standard deviation of the relative eTE, respectively. n = 6–9. * P<0.05 (Mann-Whitney U test).

Previous studies have shown that eTE can be significantly reduced in bacterial and Chinese hamster ovary (CHO) cells if they are treated with DNase within a few seconds or ∼1 min, respectively, of pulsed electric field application [33]. The observation suggests that, at these time points, either the cell membrane was still permeable to DNase resulting in degradation of internalized pDNA, or a large fraction of pDNA molecules was not internalized shortly after electric field treatment. To investigate when pDNA internalization was fully completed, we treated cells with DNase (10 U per µg pDNA for 30 min at 37°C) to digest extracellular YOYO 1-labeled pDNA or trypsin (0.25% for 30 min at 37°C) to cleave DNA-bound proteins associated with the membrane, at 10 or 40 min after electric field application. The DNase treatment was inadequate in digesting membrane-bound pDNA, as determined by fluorescence microscopy. However, pDNA bound to the membrane could be effectively removed by trypsin (see **Figure 3A**). Trypsin treatment was also able to significantly reduce eTE if administered at 10 min but had no effect at 40 min, when compared to the control (i.e., no trypsin case) (see **Figure 3B**), suggesting that the internalization of membrane-bound pDNA was completed between 10 and 40 min after electric field application.

### Effects of endocytic inhibitors on electrotransfection efficiency

**Figure 4:**
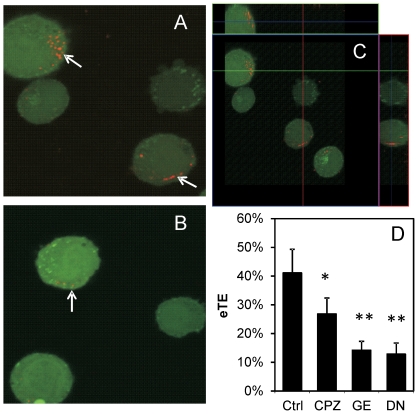
Reduction in cellular uptake of pDNA and the eTE by endocytic inhibitor treatment. pDNA covalently labeled with rhodamine (red) was electrotransfected (400 V/cm, 5 msec, 8 pulses, 1 Hz) into cells pre-treated with (A) DMSO (drug vehicle) or (B) dynasore (80 µM) for 1 hr. After electrotransfection, the cells were incubated at 37°C to enable cellular uptake of pDNA for 30 min. At the end of incubation, the cells were examined using confocal microscopy. Arrows in the microscopic images denote pDNA internalized by cells. To visualize three-dimensional distribution of pDNA in the cytosol, two optical cross-sections of DMSO-treated cells in x–z and y–z planes are shown in Panel (C). Effects of endocytic inhibitor treatment on the eTE are shown in Panel (D). Cells were treated with DMSO (Ctrl), 28 µM CPZ, 200 µM genistein (GE), or 80 µM dynasore (DN) for 1 hr prior to electrotransfection with the GFP-encoding pDNA. The eTE, defined as the percent of live cells expressing GFP, was quantified after cells were cultured at 37°C for 24 hr. n = 4–6. * P<0.05 and ** P<0.005 (Mann-Whitney U test).

Three pharmacological inhibitors of endocytosis were used in the study: chlorpromazine (CPZ), genistein, and dynasore, which block clathrin-coated pit formation, caveolae-mediated endocytosis, and dynamin activity, respectively [34], [35], [36]. Dynamin, a GTPase involved in clathrin-mediated as well as certain clathrin-independent endocytosis, facilitates fission of vesicles from the plasma membrane, resulting in release of the vesicles into the cytosol [37]. In the first experiment, B16.F10 cells were treated with dynasore or the drug vehicle DMSO (control) for 1 hr prior to electrotransfection with rhodamine-labeled pDNA. After electrotransfection, cells were incubated at 37°C for 30 min and the internalized pDNA molecules were visualized using confocal microscopy. **Figures 4A** through **4C** demonstrated qualitatively that dynasore treatment could reduce the uptake and intracellular distribution of electrotransfected pDNA in cells. In the second experiment, B16.F10 cells were pre-treated with each of the pharmacological inhibitors for 1 hr followed by electrotransfection with unlabeled pDNA encoding GFP. The eTE was quantified after 24 hr of incubation. The data shown in **Figure 4D** demonstrated that all three pharmacological inhibitors could significantly reduce eTE (P<0.05), compared to the control group, in which the cells were treated with equivalent volumes of DMSO alone.

### Dependence of electrotransfection efficiency on dynamin expression

**Figure 5:**
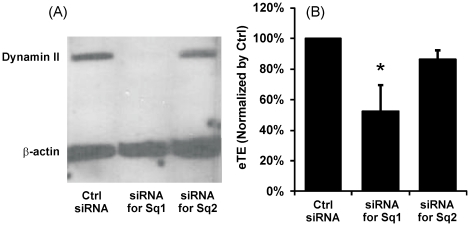
Effects of dynamin II knockdown on pDNA electrotransfection. B16-F10 cells were transfected with either the control siRNA or one of the two specific siRNA oligos directed against two different sequences (i.e., Sq1 and Sq2) in mouse dynamin II gene for silencing its expression. The siRNA treatment was followed by a 48 hr incubation period prior to pDNA electrotransfection. Dynamin II and β-actin (loading control) expression levels in Western blot analysis are shown in Panel A and normalized electrotransfection efficiencies are shown in Panel B. The bars and error bars indicate the means and standard deviations of 4 independent trials, respectively. The data from each trial, used in mean and standard deviation calculation, was the average value of replicates or triplicates. *, P<0.05 (Mann-Whitney U test).

B16-F10 cells were treated with either of two specific, small interfering RNA (siRNA) sequences directed against two different sequences in the mouse dynamin II gene (i.e., Sq1 and Sq2 described in the Materials and Methods section) or a negative control siRNA sequence with comparable GC content. Western blot analysis revealed that only siRNA sequence Sq1 resulted in sustained silencing of dynamin II expression (see **Figure 5A**) during the entire experimental period. The effects of dynamin II silencing on eTE of GFP-encoding pDNA are shown in **Figure 5B**. Cells treated with siRNA sequence Sq1 had significantly lower eTE than the control siRNA-treated cells (P<0.05). However, there was no statistically significant difference in eTE (P = 0.19) between cells treated with siRNA sequence Sq2 and the control siRNA. These data demonstrated that specific knockdown of dynamin II expression directly resulted in a ∼50% reduction in eTE.

## Discussion

Data in this study showed that Ca^2+^ and Mg^2+^ could facilitate pDNA adsorption to the cell membrane. There existed optimal concentrations of Mg^2+^ at which the percentages of pDNA-associated cells and GFP-expressing cells reached their maximum levels even though the average concentration of membrane-bound pDNA in these cells increased linearly with increasing Mg^2+^ concentration. Furthermore, the eTE could be reduced by trypsinization of cells at 10 min post electrotransfection or treatment of cells with (i) pharmacological inhibitors of endocytosis or (ii) anti-dynamin II siRNA prior to electrotransfection. These observations demonstrated that electric field-mediated cellular uptake of pDNA has three unique features: (i) it is a slow process relative to the half-life of transient electropores induced in the membrane; (ii) it must be preceded by binding of the pDNA to the plasma membrane; and (iii) it is highly dependent on specific molecules implicated in endocytic vesicle formation.

### Effects of cations on pDNA delivery

Divalent cations can affect pDNA transport into cells and electrotransfection efficiency in multiple ways. First, the cations can function as bridges that crosslink the polyanionic DNA and negatively charged carbohydrates and proteins associated with the plasma membrane of cells [1], [18], [38], [39], [40]. Ca^2+^ has also been shown to form ternary complexes with DNA and negatively charged lipids in unilamellar liposomes [39]. The amount of DNA adsorbed on the lipid membrane increases with increasing concentration of cations [38]. In our study, the percent of pDNA-associated cells reached a maximum value of <16% (see **Figure 1A**) whereas the amount of pDNA adsorbed to these cells continued to increase linearly with increasing concentration of cations (see **Figure 1B**). One possible explanation may be that the membrane-associated carbohydrates and proteins in >80% of the cells might have been thoroughly stripped off by the trypsin treatment required to generate the single-cell suspensions needed in this study. Therefore, pDNA molecules could not adsorb to the majority of cells. Another observation shown in **Figure 1** was that the amount of pDNA adsorbed on the membrane was low but not zero when no divalent cations were present, suggesting that non-specific binding between pDNA and the membrane was present, albeit weak. It should be pointed out that pDNA adsorption to the membrane via divalent cations differs from the complex formation between pDNA and the membrane discovered in previous studies [22], [23], [24], [41]. The latter is induced by pulsed electric field whereas the former is field-independent. However, the cation-mediated adsorption can enhance complex formation by increasing the local concentration of pDNA next to the cell membrane. The enhancement might explain why the peaks of eTE, shown in **Figure 2A**, occurred at lower concentrations of cations, compared to those of percent of pDNA-associated cells (see **Figure 1A**).

Second, divalent cations in the electrotransfection buffer can bind to pDNA to neutralize its charge and cause DNA aggregation [42]. The neutralization will reduce the driving force for electrophoresis during electrotransfection, thereby reducing pDNA transport from extracellular medium into cells. At higher concentrations, divalent cations could cause pDNA aggregation by cross-linking multiple DNA molecules. Both neutralization and aggregation can reduce the eTE since less pDNA molecules will be delivered into cells. These phenomena may explain the trend observed in some previous studies where eTE decreased with increasing concentration of Mg^2+^ [1], [18]. However, our data shown in **Figure 2A** differ from these studies, in that the eTE exhibits a bell-shaped dependence on both the Ca^2+^ and Mg^2+^ concentrations. Our data are similar to the results reported in another study [38]. The discrepancy between these studies could be caused by differences in experimental design. In the study by Haberl et al. [18], pDNA was added into the electrotransfection buffer (40 µg/ml), containing cells (2.5×10^6^ per ml) and Mg^2+^, within 2–3 min before pulsed electric field treatment. In our study, the pDNA concentration was 50% higher, the cell density was 4 times higher, and pDNA was added into the buffer 5–10 min before electric field application. The increase in both pDNA concentration and cell density reduced the average distance between pDNA and cells. With the shorter diffusion distance and longer diffusion time, our protocol allowed more pDNA molecules to be adsorbed on the membrane before electrotransfection. As a result, pDNA delivery towards cells was less reliant on electrophoresis during electrotransfection since a significant fraction of pDNA molecules were already bound to the membrane before exposure to electric field [38]. However, higher cation concentrations may result in a higher binding affinity between pDNA and membrane such that pDNA remains anchored to the membrane after electric field application, which makes pDNA unable to enter the cell for subsequent GFP expression [18]. pDNA immobilization might be one of the factors contributing to the decrease in eTE observed in **Figure 2A** when Ca^2+^ or Mg^2+^ concentration exceeded a threshold level. It should be noted that the low ionic strength buffer supplemented with Ca^2+^ or Mg^2+^ was not optimized for electrotransfection since it contained only one cation, Ca^2+^ or Mg^2+^. In other studies, OptiMEM is used as electrotransfection buffer, which likely contains several multivalent cations and other charged species that could enhance the binding of DNA to the cell membrane although the exact formulation of OptiMEM is unknown due to its proprietary nature. The differences in cation species and their concentrations may explain why the peak levels of eTE in our buffer were lower than the average eTE in OptiMEM (see **Figure 2**). It is expected that the new mechanisms of electrotransfection revealed in this study may lead to development of new, optimized buffers that may outperform OptiMEM in terms of eTE.

Third, it is well known that Ca^2+^ and Mg^2+^ can enhance exocytosis, endocytosis, and vesicle recycling [37], [43], [44], [45], [46], which are often coupled together in cells. Extracellular concentrations of these cations used in the previous studies vary between 1 and 10 mM [43], [45], [46], which were similar to the concentrations used in our study. Fourth, endonuclease activity can be enhanced by divalent cations at 1 mM concentration [28], which may reduce the half-life of DNA in cells. However, this effect is negligible in our study as shown by the data in **Figures 2B and 2C**, in which cations delivered into cells shortly after electrotransfection had minimal effects on the eTE. Data in the literature and our study combined suggest that Ca^2+^ and Mg^2+^ could potentially affect pDNA transport into cells and the eTE by facilitating pDNA binding to cell membrane and enhancing endocytosis of membrane-bound pDNA.

### Effects of adsorbed pDNA on membrane structures

A flexible membrane is required for invagination and internalization of bound pDNA molecules. There is evidence that pDNA adsorption may decrease membrane stiffness and cause structural changes in the membrane. When small DNA fragments (∼300 bp) are adsorbed to the membrane of unilamellar vesicles via Ca^2+^, the membrane stiffness is decreased, which reduces the energy barrier to electroporation [47]. Similarly, large T7 DNA and pDNA molecules adsorbed onto lipid membrane through divalent cations can induce formation of DNA-containing endosome-like vesicles in unilamellar liposomes [48]. The liposomal uptake of large DNA can be further enhanced by applying pulsed electric field [48]. The same structural changes might also occur in the plasma membrane of living cells to facilitate the endocytosis of pDNA observed in the study, although further investigation is required to demonstrate these changes directly.

### Dynamics of pDNA internalization after exposure to electric field

DNA visualization studies demonstrate the formation of both metastable and stable complexes between pDNA and the region of the plasma membrane facing the cathode, when the field magnitude exceeds a critical threshold [22], [23], [24], [41]. It is the stable form of these complexes, which cannot be eliminated by reversing the polarity of the electric field, that determines the efficiency of gene delivery. pDNA molecules in the complex are susceptible to external staining with TOTO-1 dye at 1 sec after pulsing but become inaccessible to TOTO-1 at 10 min [23]. In another study, the pDNA molecules are accessible to external DNase within 1 min after the application of electric field [33]. These findings, albeit important, do not clearly determine the location of the pDNA complexes as being inside the cell, within the membrane, or on the extracellular side of the membrane since the lack of fluorescence staining or pDNA degradation may be attributed to an inability of TOTO-1 or DNase to penetrate the complexes and access their target DNA sites. Data in this study suggested that a significant fraction of the pDNA complexes were still on the outer surface of the membrane at 10 min post electrotransfection since trypsinization of cells could reduce the eTE (see **Figure 3**). At 40 min following electrotransfection, trypsinization had no effects on the eTE, suggesting that the process of cellular uptake of pDNA was completely finished between 10 and 40 min, which greatly exceeds the lifetime (∼10 msec) of the transient membrane pores induced by electric field [11]. This data implies that a large fraction of pDNA molecules entered the cells after the pores had resealed, which precludes diffusion and electrophoresis from playing important roles in transmembrane transport of pDNA molecules.

### Effects of endocytic impairment on electrotransfection efficiency

In this study, we demonstrated statistically significant reductions in the eTE induced by different pharmacological inhibitors of endocytosis (i.e., chlorpromazine, genistein, and dynasore) (see **Figure 4D**). It was also observed in **Figure 4A–C** that dynasore substantially reduced the intracellular distribution of rhodamine-labeled pDNA following electric field treatment, which explains the corresponding reduction in eTE shown in **Figure 4D**. These findings were further substantiated by the siRNA silencing experiment, which showed a substantial reduction in eTE as a direct result of dynamin II knockdown (**Figure 5**). These data together strongly suggested that electric field mediated-pDNA uptake and intracellular transport relied, in some capacity, on endocytic pathways and vesicle trafficking mechanisms. Further investigations are required to understand the specific endocytic pathways and mechanisms involved.

Several previous studies have speculated upon or presented indirect evidence of the potential role of endocytosis in electrotransfection. For example, it is energetically feasible for the applied electric field to induce membrane invagination during electrotransformation [49], [50]. Pulsed electric field has been observed to enhance liposomal uptake of membrane bound DNA into endosome-like vesicles [48]. For mammalian cells, Favard *et al.* have observed the size of these electric field induced pDNA-membrane complexes to be on the same order of magnitude as that of lipid rafts [24], [27], which are plasma membrane microdomains enriched in cholesterol and sphingolipids that are involved in raft-dependent endocytosis [51], [52], [53], [54]. Direct measurements showed that pulsed electric field could stimulate endocytosis of fluorescein-label bovine serum albumin [55], [56], [57] and β-galactosidase [58].

A seemingly contradictory study was conducted by Pavlin et al., in which the authors observed that both high and low electric field could lead to vesicle formation within mammalian cells but only high electric field would result in successful gene delivery [59]. Thus, the authors concluded that endocytosis of pDNA was not involved in electrotransfection and suggested that the vesicles induced by the applied electric field were related to exocytosis that was triggered for repairing electrically-damaged membrane. However, an alternative explanation for the same observation exists. The endosome-like vesicle formation has been observed in unilamellar liposomes when liposomes in the DNA solution are exposed to pulsed electric field [48]. In this case, there is no exocytosis. The vesicles formed have facilitated DNA uptake by liposomes and the amount of uptake increases with increasing electric field strength [48]. Furthermore, it has been shown that DNA-membrane complex formation is a necessary step for electrotransfection and that complex formation occurs only if the field magnitude exceeds a threshold [22], [23], [24], [41]. Therefore, the lack of transgene expression at low electric field observed by Pavlin et al. could be due to the inability of pDNA molecules to form stable complexes with the cell membrane.

It has become a widely accepted, yet poorly proven hypothesis that electroporation delivers macromolecules, regardless of their nature, directly into the cytosol via diffusion and/or electrophoresis through transient pores created by pulsed electric field. The findings in this study offer the first compelling, direct evidence to support an alternative hypothesis that electric field-mediated internalization of pDNA is dependent upon endocytic pathways. Data in this study show that (a) the internalization process was several orders of magnitude longer than the known lifetime of transient pores induced by electric field, (b) pDNA adsorption to the plasma membrane was a necessary step for cellular uptake during electrotransfection, and (c) both cellular uptake of pDNA and transgene expression could be reduced by using well-established endocytic inhibitors and anti-dynamin II siRNA [34], [35], [36]. The endocytosis can be triggered by pulsed electric field both directly [48], [55], [56], [57], [58], [60] and indirectly through inducing the formation of pDNA-membrane complexes [22], [23], [24]. Specific pathways of pDNA endocytosis remain unknown and will need to be investigated in future studies. An understanding of these pathways will allow scientists to develop novel strategies for improving efficiencies of cell transfection and gene manipulation.

## Materials and Methods

### Cell culture

B16.F10, a murine melanoma cell line [61], [62], [63], were cultured as monolayers in T75 flasks in high glucose Dulbecco's modified Eagle's medium (Invitrogen, Carlsbad, CA) supplemented with 10% bovine growth serum (Hyclone, Logan, UT) and penicillin/streptomycin (Invitrogen). The cells were incubated at 37°C in 5% CO_2_ and 95% air and passaged every 2–3 days. For adsorption and electrotransfection experiments, 75–90% confluent T75 flasks were treated with 0.25% trypsin-EDTA (Invitrogen) for 5 min at 37°C, harvested by centrifugation, and washed with serum-containing medium to neutralize the activity of trypsin. Cells were then washed again with an electrotransfection buffer before final re-suspension in the same buffer at a concentration of 0.5–1×10^7^ cells/ml. Two electrotransfection buffers were used in the study: 1) OptiMEM I Reduced Serum Media (Invitrogen, Carlsbad, CA) and 2) a low ionic strength medium (250 mM sucrose, 20 mM HEPES) supplemented with varying concentrations of calcium or magnesium ranging from 0.1–30 mM.

### Plasmid

A 4.7 kbp plasmid (pEGFP-N1, Clontech, Palo Alto, CA), encoding enhanced green fluorescent protein (GFP), was amplified by transformed, Z-competent (Zymo Research, Orange, CA) DH5α and Top10 *E. coli* strains and purified using the Qiagen Plasmid Maxi Prep Kit (Qiagen, Valencia, CA), as per manufacturers' protocols. For visualization and adsorption studies, the plasmid DNA (pDNA) was labeled with either YOYO-1, a DNA-intercalating fluorescence dye (Invitrogen), or LabelIT tetramethylrhodamine (TMR), which covalently binds with DNA (Mirus Corp., Madison, WI). Depending on the concentration of pDNA, an appropriate volume of the 1 mM stock YOYO-1 solution was mixed with the pDNA solution to yield a basepair-to-dye ratio of 5∶1. The mixture was incubated at room temperature for at least 60 min prior to use. Covalent TMR labeling of the pDNA using the LabelIT kit was conducted as per manufacturer's protocol.

### Membrane adsorption of plasmid DNA

To study pDNA adsorption to the plasma membrane mediated by the cations (Ca^2+^ and Mg^2+^), 3 µg of YOYO-1 labeled pDNA was added to 1 million B16-F10 cells suspended in 200 µL of the low ionic strength buffer with varying concentrations of Ca^2+^ or Mg^2+^. The samples were incubated for 10 min on ice followed by 10 min at room temperature to simulate the electotransfection protocol without actual pulse application. Samples were then pelleted via centrifugation and re-suspended in 350–400 µL PBS without Ca^2+^ and Mg^2+^. The percentage of cells that were associated with pDNA as well as the average fluorescence intensity of the same population of cells were quantified using flow cytometry.

### Trypsin-mediated removal of membrane-adsorbed pDNA

To remove electric field-induced, membrane-adsorbed pDNA, B16-F10 cells were subjected to trypsin treatment following electrotransfection. In the experiment, cells were treated with 0.25% trypsin-EDTA for 30 min at 37°C, starting at 10 min post electrotransfection, which allowed cell recovery and electric field induced transient pores in the membrane to re-seal at room temperature. Following the trypsin-EDTA treatment, the cells were washed with serum-rich DMEM to neutralize the trypsin, seeded in fresh serum-rich DMEM in 6-well plates, and incubated for 24 hr. Flow cytometry was used to assess the effect of trypsin treatment on GFP expression.

### Electrotransfection

1 million B16-F10 cells suspended in electrotransfection buffer (either OptiMEM or low ionic strength buffer supplemented with Ca^2+^ or Mg^2+^) were mixed with 6 µg pEGFP-N1 to achieve a final sample volume of 100 µL. Samples were loaded into BTX disposable 4-mm gap aluminum cuvettes (Harvard Apparatus, Holliston, MA), and incubated on ice for 5–10 min before receiving an electric treatment of 8 pulses at 400 V/cm, 5 msec duration, and 1 Hz frequency. The pulses were generated by using the BTX ECM 830 Square Wave Electroporation System (Harvard Apparatus). Samples were left in the cuvette for 10 min at room temperature post electrotransfection to promote cell recovery and resealing of pores in the membrane. Then, the samples were spun down, decanted of electrotransfection buffer, and seeded in fresh serum-rich DMEM in 6-well plates. GFP expression was quantified using flow cytometry following 24 hr of incubation.

To determine if the cations at varying concentrations could alter GFP expression, aside from mediating membrane adsorption of pDNA, 2 million B16-F10 cells were mixed with 12 µg pEGFP-N1 in 200 µL of OptiMEM, incubated on ice for 5 min, and treated electrically using the same pulsing protocol as mentioned above. Samples were left in the cuvette for 20 min at room temperature to ensure complete resealing of pores in the membrane, spun down, and re-suspended in 200 µL of low ionic strength buffer of varying Ca^2+^ or Mg^2+^ concentrations. The cell suspensions were incubated on ice for 5 min, treated with the same electric field as above, and left in the cuvette for another 10 min at room temperature. Then, the cells were seeded in fresh serum-rich DMEM and GFP expression was quantified after 24 hr of incubation at 37°C.

### Treatment of cells with pharmacological inhibitors of endocytosis

Stock solutions of the endocytic inhibiting drugs, chlorpromazine (CPZ), genistein, and dynasore (Sigma Aldrich, St. Louis, MO), were prepared in DMSO at concentrations of 5 mg/ml, 25 mg/ml, and 10 mg/ml, respectively, and stored at −20°C. 7 to 8×10^5^ cells were seeded per well in a 6-well plate overnight to achieve 75–90% confluency in the following day. Media was aspirated and adherent cells were washed twice with PBS without Ca^2+^ and Mg^2+^. 2 mL of serum-free DMEM was added to each well and appropriate volumes of the drugs were added to achieve final drug concentrations of 28 µM, 200 µM, and 80 µM for CPZ, genistein, and dynasore, respectively. In the control groups, equivalent volumes of DMSO without drugs were added into the wells. Cells were incubated with the drugs or DMSO at 37°C in 5% CO_2_ and 95% air for 1 hr and subsequently washed with PBS without Ca^2+^ and Mg^2+^. Cells were trypsinized, washed again with serum-containing media, and re-suspended in OptiMEM at a density of 1×10^7^ cell/mL. They were subsequently electrotransfected with pDNA encoding GFP to investigate effects of the drug treatment on electrotransfection efficiency.

### Flow cytometry

DMEM was aspirated from each well and adherent cells were washed twice with PBS without Ca^2+^ and Mg^2+^. The cells were then detached by trypsinization, washed with serum-rich DMEM to neutralize trypsin activity, and resuspended in 350–400 µL of PBS containing propidium iodide (PI) (5 µg/mL). The BD FACScan flow cytometer (Becton Dickinson, Franklin Lakes, NJ), equipped with an argon laser with excitation wavelength of 488 nm and three fluorescence detectors (530/30, 585/42, 670LP), was used for simultaneous dual detection of GFP and PI fluorescence. Forward and side light scatterings were used as independent variables to exclude debris and isolate the cell population of interest. Compensation was set between 20–25% to resolve spectral emission overlap between the two channels and 10,000 events were collected for each sample. Autofluorescence from cells was corrected in each experimental group by using cell samples treated with the same protocol but in the absence of pDNA. BD CELLQUEST™ Software was used for data acquisition and analysis. The electrotransfection efficiency was expressed as the percentage of total viable cells expressing GFP (PI negative, GFP positive).

### Knockdown of dynamin II expression

B16-F10 cells were transfected with either of two specific Stealth small interfering RNA (siRNA) oligos (Invitrogen) directed against mouse dynamin II gene or the Stealth negative control siRNA (Medium GC) duplex (Invitrogen) using the Amaxa Nucleofector II System (Amaxa Biosystems, Cologne, Germany). The two siRNA oligos targeted the following sequences in the mouse dynamin II gene: Sq1: 5′-GAGCCCGCATCAATCGTATCTTTCA-3′ and Sq2: 5′-CATGAGCTGCTGGCTTACCTGTATT-3′.

In the experiment, 1.5×10^6^ cells were transfected with one of the siRNA oligos at a dose of 30 pmols using the Nucleofector Kit V (Amaxa) and Program P-020. The transfected cells were distributed evenly into 4 wells in a 6-well plate in order to achieve ∼90% confluency after 48 hr incubation. Cells were then harvested for Western blot analysis and pDNA electrotransfection experiment.

### Western blot analysis

B16-F10 cells were subjected to siRNA treatment and pDNA electrotransfection in the same manner as described above, with the exception that all constituent quantities/volumes were tripled for electrotransfection to ensure sufficient protein yield. Dynamin II expression levels were detected via immunoblot analysis after a 24-hr post-electrotransfection incubation period. Total protein content was isolated using a 10∶1 mixture of CellLytic M Cell Lysis Reagent (Sigma Aldrich) to Protease inhibitor cocktail (Sigma Aldrich) and quantified using Pierce BCA Protein Assay Kit (Thermo Scientific, Rockford, IL). 10 µg of total protein extract per well was separated in a 7.5% Ready Gel Tris-HCl polyacrylamide gel (Bio-Rad, Hercules, CA), transferred onto PVDF membrane (Bio-Rad), blocked for 2–3 hours at room temperature in 5% milk, 20 mM Tris, 500 mM NaCl, 0.1% Tween20 (pH 7.5) and incubated overnight at 4°C with primary antibodies: mouse anti-dynamin II (B-2) (Santa Cruz Biotech, Santa Cuz, CA) and mouse anti-β-actin (C4) (Santa Cruz) as a loading control. Membrane was then probed with goat anti-mouse secondary antibody conjugated to horseradish peroxidase (Santa Cruz) for 45 min at room temperature, developed with SuperSignal West Pico Chemiluminescent Substrate (Thermo Scientific), and visualized with Amersham Hyperfilm™ ECL film (GE Healthcare, Buckinghamshire, UK).

### Confocal microscopy

All fluorescence images were acquired using a confocal laser scanning microscope (LSM510, Carl Zeiss, Thornwood, NY) equipped with a 100× oil-immersion objective. Images shown in the figures represent optical slices near the middle plane of cells.

### Statistical analysis

Difference between two experimental groups was compared using the Mann-Whitney U test. A difference was considered to be significant if the P value was <0.05.
